# Mitochondrial protein sorting as a therapeutic target for ATP synthase disorders

**DOI:** 10.1038/ncomms6585

**Published:** 2014-12-18

**Authors:** Raeka S. Aiyar, Maria Bohnert, Stéphane Duvezin-Caubet, Cécile Voisset, Julien Gagneur, Emilie S. Fritsch, Elodie Couplan, Karina von der Malsburg, Charlotta Funaya, Flavie Soubigou, Florence Courtin, Sundari Suresh, Roza Kucharczyk, Justine Evrard, Claude Antony, Robert P. St.Onge, Marc Blondel, Jean-Paul di Rago, Martin van der Laan, Lars M. Steinmetz

**Affiliations:** 1 European Molecular Biology Laboratory (EMBL), Genome Biology Unit, 69117 Heidelberg, Germany; 2 Institut für Biochemie und Molekularbiologie, ZBMZ, Universität Freiburg, 79104 Freiburg, Germany; 3 Université Bordeaux, IBGC, UMR 5095, F-33000 Bordeaux, France; 4 CNRS, IBGC, UMR 5095, F-33000 Bordeaux, France; 5 Institut National de la Santé et de la Recherche Médicale UMR1078; Université de Bretagne Occidentale, Faculté de Médecine et des Sciences de la Santé; Etablissement Français du Sang (EFS) Bretagne; CHRU Brest, Hôpital Morvan, Laboratoire de Génétique Moléculaire, Brest F-29200, France; 6 BIOSS Centre for Biological Signalling Studies, Universität Freiburg, 79104 Freiburg, Germany; 7 European Molecular Biology Laboratory (EMBL), Electron Microscopy Core Facility, 69117 Heidelberg, Germany; 8 Stanford Genome Technology Center, Stanford University, Palo Alto, California 94304, USA; 9 Department of Genetics, Institute of Biochemistry and Biophysics, Polish Academy of Sciences, Warsaw, Poland; 10 Department of Genetics, Stanford University School of Medicine, Stanford, California, USA

## Abstract

Mitochondrial diseases are systemic, prevalent and often fatal; yet treatments remain scarce. Identifying molecular intervention points that can be therapeutically targeted remains a major challenge, which we confronted via a screening assay we developed. Using yeast models of mitochondrial ATP synthase disorders, we screened a drug repurposing library, and applied genomic and biochemical techniques to identify pathways of interest. Here we demonstrate that modulating the sorting of nuclear-encoded proteins into mitochondria, mediated by the TIM23 complex, proves therapeutic in both yeast and patient-derived cells exhibiting ATP synthase deficiency. Targeting TIM23-dependent protein sorting improves an array of phenotypes associated with ATP synthase disorders, including biogenesis and activity of the oxidative phosphorylation machinery. Our study establishes mitochondrial protein sorting as an intervention point for ATP synthase disorders, and because of the central role of this pathway in mitochondrial biogenesis, it holds broad value for the treatment of mitochondrial diseases.

## Abstract (web summary)

Effective treatment options for mitochondrial diseases are scarce. Here, Aiyar *et al*. identify the TIM23 mitochondrial protein sorting machinery as a potential intervention point for mitochondrial ATP synthase disorders.

## Graphical abstract

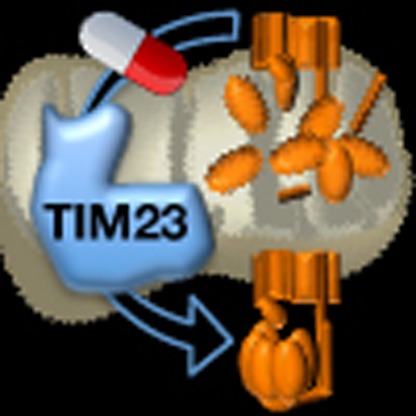

Mitochondrial dysfunction has been implicated in a host of diseases with a wide range of symptoms and molecular phenotypes, which present significant challenges to diagnosis, let alone treatment^1^,^2^,^3^,^4^. Many of these disorders affect mitochondrial ATP synthase, which produces most of the cellular ATP required in humans through the process of oxidative phosphorylation (OXPHOS). Defects in the structure or assembly of this crucial enzyme result in severe diseases that manifest primarily in children, often shortly after birth. Various ATP synthase disorders resulting from defects in the structure or assembly of this enzyme complex have been described, including neuropathy, ataxia and retinitis pigmentosa (NARP), a fatal encephalopathy known as Leigh syndrome, and hypertrophic cardiomyopathy^5^,^6^,^7^. Many of the molecular consequences of these disorders, ranging from deficient ATP production to widespread effects on mitochondrial biogenesis, structure and metabolism have been studied using yeast models, due to the extensive conservation of mitochondrial functions and the ability to manipulate the yeast mitochondrial and nuclear genomes^7^.

One relevant model of ATP synthase disorders is a strain lacking Fmc1 (*fmc1*Δ), a factor required for the assembly of the F_1_ sector of ATP synthase at high temperatures^8^. Loss of Fmc1 directly and severely impairs ATP synthase assembly, while secondarily impairing the respiratory chain biogenesis and activity as well as mitochondrial membrane potential^9^. These secondary consequences on the respiratory chain are frequently observed in the context of reduced ATP synthase activity^10^,^11^,^12^,^13^, and are believed to reflect a regulatory mechanism that maintains a balanced production of ATP synthase and respiratory chain complexes^7^,^12^,^14^. Because of these deficiencies, *fmc1*Δ yeast display impaired growth on non-fermentable carbon sources like glycerol. Their drastic reduction in fully assembled ATP synthase along with their impaired mitochondrial respiration result in 90% less mitochondrial ATP synthesis^8^,^9^. Since these phenotypes are also found in multiple patients^15^,^16^, we have leveraged this model system to develop a stringent, high-throughput screening assay for the identification of compounds that suppress ATP synthase deficiency^14^.

In this study, we apply our screening assay to search for pathways that can be therapeutically targeted for the treatment of ATP synthase disorders. By following up one of the hits using chemical–genomic analysis, we identify the major mitochondrial protein sorting pathway mediated by the inner membrane presequence translocase (TIM23 complex) as a potential intervention point for these disorders. We show that a specific modulation of this pathway, which we recapitulate using a genetic modification, is sufficient to rescue several phenotypes associated with ATP synthase deficiency. Notably, modulating this pathway is also effective in cells derived from ATP synthase-disorder patients, demonstrating the conservation of these therapeutic effects. Our study thus provides the first indication that mitochondrial protein sorting is a promising therapeutic target for the treatment of ATP synthase disorders.

## Results and discussion

### Sodium pyrithione improves growth of ATP synthase-deficient cells

**Figure 1:**
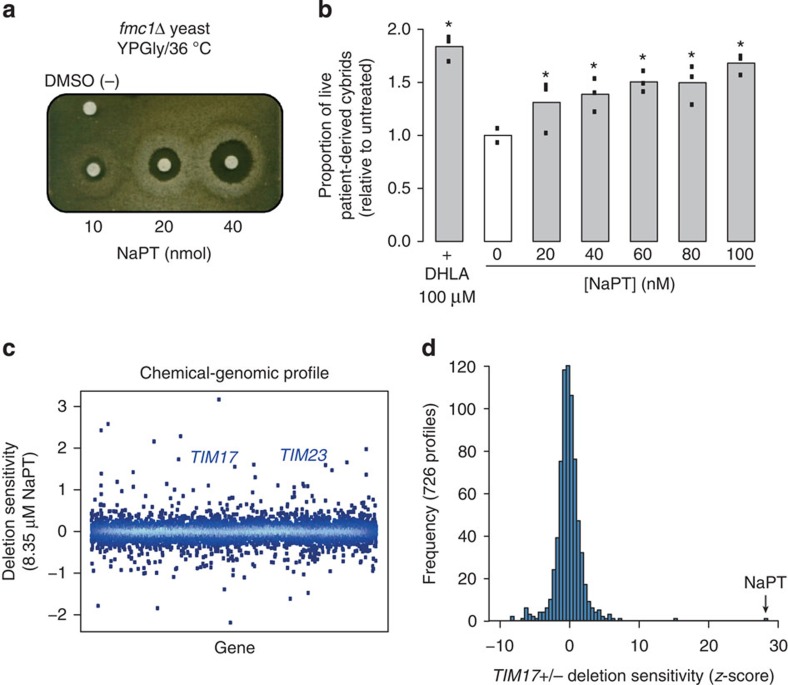
Identification of NaPT in a drug screen and a potential new therapeutic target revealed by a genomic screen. (**a**) NaPT rescues respiratory growth of the *fmc1*Δ mutant, a yeast model for ATP synthase disorders, in a dose-dependent manner. *fmc1*Δ cells were spread onto solid rich glycerol medium, indicated amounts of NaPT were spotted onto filters, and plates were incubated at 36 °C for 6 days. High concentrations of NaPT are toxic, as indicated by the regions with no growth immediately surrounding the filters. The halos of growth at dose-dependent distances from the filters correspond to the lower therapeutic concentrations. DMSO (−), compound vehicle. (**b**) NaPT treatment improves survival of *atp6-T8993G* (JCP239) cybrids derived from ATP synthase disorder patients in glucose-deprived medium in a dose-dependent manner. Data from three replicates per condition are indicated by points; height of the bar represents the mean. Statistical significance: *P<0.05, (Wilcoxon’s test) relative to the untreated sample (0). DHLA, positive control, drug currently in clinical trials for mitochondrial encephalopathies^14^,^18^. (**c**) The chemical–genomic profile of NaPT identifies mitochondrial protein sorting as a potential target. On treatment of the yeast genome-wide deletion collection with 8.35 μM NaPT, *TIM17* and *TIM23*, essential genes involved in mitochondrial protein import, displayed pronounced deletion sensitivity/haploinsufficiency (calculated relative to the same strain collection treated with DMSO as a negative control; see Methods). Increasing data density is depicted as lighter shades of blue. (**d**) *TIM17*-deletion sensitivity is specific to NaPT. Frequency distribution of the *TIM17*^+/*−*^ mutant *z*-scores (see Methods) from 726 published chemical–genomic profiles^23^ across 332 compounds compared with the score obtained for NaPT.

From a drug repurposing library^17^, our screening assay identified sodium pyrithione (NaPT), a small molecule currently used as an antiseptic. NaPT markedly improved the respiratory growth of *fmc1*Δ yeast in a dose-dependent manner (Fig. 1a). Notably, this compound was also effective in a cybrid (cytoplasmic hybrid) cell line derived from NARP patients carrying the *atp6-T8993G* mutation, a mutation also implicated in maternally inherited Leigh’s syndrome^7^,^18^. This mutation severely perturbs OXPHOS in both patient tissues and cells derived from them, resulting in extremely poor survival of the latter in glucose-deprived medium^18^,^19^,^20^. Treatment with NaPT improved the survival of *atp6-T8993G* cybrids, in a dose-dependent manner, up to 1.8-fold, but did not affect control cybrids (Fig. 1b; Supplementary Fig. 1a; Supplementary Data Set 1). These data suggest that NaPT rescues ATP synthase deficiency via a pathway that is conserved between yeast and humans.

### Chemical-genomic profiling of NaPT-treated cells

To identify the pathway(s) through which NaPT could be rescuing ATP synthase deficiencies, we carried out systematic chemical–genomic profiling using the yeast genome-wide deletion collection (Fig. 1c; Supplementary Data Set 2). In this approach, pronounced sensitivity of heterozygous deletion mutants (that is, haploinsufficiency) to inhibitory concentrations of a chemical indicates the genes and pathways involved in the chemical’s mechanism of action^21^,^22^,^23^. The 10 deletion mutants most sensitive to inhibitory concentrations of NaPT implicated two essential mitochondrial pathways: protein sorting and iron-sulfur cluster biogenesis. The latter is consistent with a previous report that the zinc salt of PT inhibits yeast growth through copper influx and inactivation of iron-sulfur cluster proteins^24^. The mitochondrial protein sorting pathway was indicated by the genes *TIM17* and *TIM23*, which encode core components of the highly conserved presequence translocase of the inner mitochondrial membrane (TIM23 complex)^25^,^26^. The sensitivities of the *TIM17*^*+/−*^ and *TIM23*^*+/−*^ mutants to NaPT were far greater than their sensitivity to hundreds of previously profiled compounds^23^ (Fig. 1d; Supplementary Fig. 1b), indicating that the chemical-genetic interaction between NaPT and the TIM23 machinery is specific.

The TIM23 complex mediates the sorting of nuclear-encoded mitochondrial proteins with amino terminal presequences as targeting signals to either the inner mitochondrial membrane or the matrix, which includes many subunits of ATP synthase and respiratory chain complexes^27^,^28^,^29^. Notably, a recent study has demonstrated that the import of respiratory chain proteins into mitochondria via TIM23 can be directly coupled to their assembly into active complexes^30^. As outlined above, the activity of these respiratory chain complexes is often perturbed in ATP synthase disorders^12^,^13^,^15^,^16^. Taken together, these observations suggested that the sorting of presequence-carrying proteins into mitochondria could be an attractive therapeutic target for ATP synthase deficiencies.

### NaPT modulates mitochondrial protein sorting via TIM23

**Figure 2:**
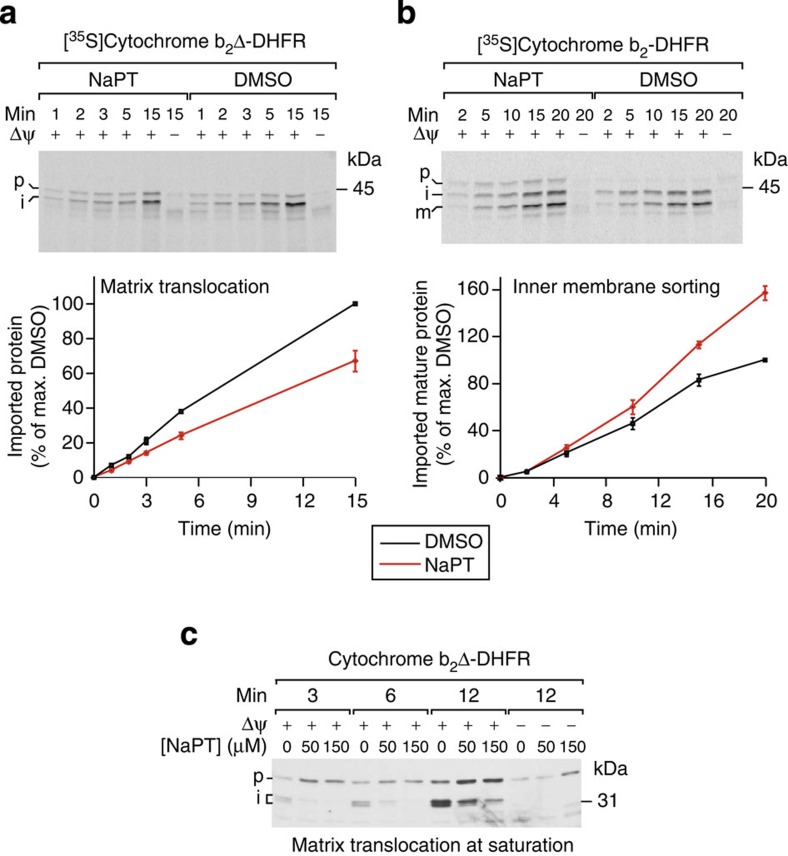
NaPT selectively and differentially modulates mitochondrial protein sorting via the presequence translocase TIM23. (**a**) Import of *in vitro*-synthesized, radiolabelled cytochrome *b*_2_Δ-DHFR, a model protein targeted to the mitochondrial matrix, into isolated yeast mitochondria (p=precursor; i=intermediate) in the presence or absence of 100 μM NaPT. The lower panel displays quantifications of the bands detected by autoradiography in three independent experiments (imported, protease-resistant i-form). The signal obtained in the presence DMSO after the longest incubation time was set to 100%. Error bars represent the s.e.m. Δψ, mitochondrial inner membrane potential. (**b**) Import of *in vitro* synthesized, radiolabelled cytochrome *b*_2_-DHFR, targeted to the inner membrane, into isolated mitochondria (m=mature). The lower panel displays quantifications of the bands (imported, protease-resistant m-form) as in **a**. (**c**) Importing saturating amounts of recombinantly expressed and purified cytochrome *b*_*2*_Δ-DHFR causes a pronounced, concentration-dependent inhibition by NaPT. Import reactions were analysed by immunoblotting using an antibody against DHFR.

The expression levels of subunits of the TIM23 complex and the presequence-translocase-associated import motor, which is essential for matrix translocation^27^,^28^,^29^, were not affected by NaPT treatment in wild-type or *fmc1*Δ cells (Supplementary Fig. 1c). Therefore, to directly test the effect of NaPT on both mitochondrial protein import activities mediated by TIM23, we carried out *in vitro* import assays using established model substrates targeted to the inner membrane (cytochrome *b*_2_-DHFR) or the matrix (cytochrome *b*_2_Δ-DHFR, lacking the membrane-sorting signal)^31^,^32^ (Fig. 2a,b; Supplementary Fig. 6; Supplementary Data Set 3). In the presence of NaPT, import of matrix-targeted cytochrome *b*_2_Δ-DHFR was significantly inhibited (33% slower, *P*<5.5 × 10^*−*13^, Fig. 2a). In contrast, import of inner membrane-targeted cytochrome *b*_2_-DHFR was enhanced (46% faster, *P*<1.3 × 10^*−*8^; Fig. 2b). When importing a saturating amount of cytochrome *b*_2_Δ-DHFR (using recombinantly expressed purified protein versus the *in vitro*-synthesized substrate above, see Methods), the impairment of matrix import by NaPT and its dose dependence were striking (Fig. 2c; Supplementary Fig. 6). We verified that NaPT does not disrupt general properties required for mitochondrial protein import, including membrane integrity and inner membrane potential (Δψ) (Supplementary Fig. 2a,b). Moreover, other major mitochondrial protein import pathways, including the assembly of β-barrel proteins into the outer membrane by the SAM (TOB) complex and insertion of proteins with internal import signals into the inner membrane by the TIM22 complex^27^,^28^, were not affected by NaPT (Supplementary Fig. 2c,d). We conclude that NaPT does not interfere with the general import competence of mitochondria, but selectively and differentially modulates import of presequence proteins via the TIM23 pathway. These findings are in line with recent studies indicating that the downregulation of mitochondrial protein import via TIM23 can be employed as a stress-responsive mechanism to maintain protein homeostasis in mitochondria^33^,^34^.

To our knowledge, this is the first report of a compound with highly specific effects on TIM23-mediated protein sorting, a central pathway of mitochondrial biogenesis. Nevertheless, we also found that some effects of NaPT on protein sorting *in vitro* were influenced by the presence of copper (Supplementary Figs. 3,7c–e), which is consistent with previous reports^24^ and could complicate both the drug’s *in vivo* effects^35^ and its viability as a drug development candidate. We therefore sought an independent approach to confirm the role of TIM23-mediated mitochondrial protein sorting in suppressing ATP synthase deficiencies.

### Increased Tim21 levels rescue ATP synthase-deficient cells

**Figure 3:**
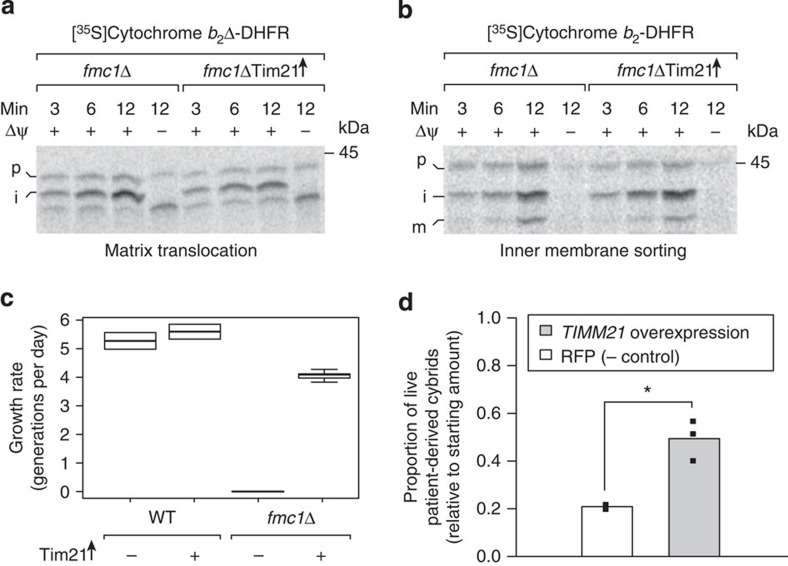
Genetic modulation of mitochondrial protein sorting rescues yeast and human ATP synthase disorder models. (**a**,**b**) Tim21 overexpression induces NaPT-like modulation of TIM23-mediated mitochondrial protein import. Assays as in Fig. 2a,b were performed with mitochondria isolated from *fmc1*Δ cells carrying Tim21 overexpression (Tim21↑) versus empty plasmids. Data are representative of three independent experiments. (**c**) Tim21 overexpression suppresses the respiratory growth defect of *fmc1*Δ yeast. Strains were grown in liquid rich glycerol media (YPG) at 36 °C until stationary phase; their maximum growth rate (in generations per day, see Methods) is displayed in a boxplot (WT (wild type), *n*=3; *fmc1*Δ, *n*=5). (**d**) Overexpression of *TIMM21*, the human ortholog of yeast *TIM21*, improves cybrid survival. NARP *atp6-T8993G* cybrids were transduced with lentiviral particles carrying *TIMM21* and GFP or RFP alone as a negative control. After 6 days, surviving cells were counted using flow cytometry. Individual replicates are displayed as points; height of the bar represents the mean; * indicates statistical significance (*P*<0.05, Wilcoxon’s test).

To study the therapeutic potential of modulating mitochondrial protein sorting in isolation, we applied a genetic modification that recapitulates the effects of NaPT on the TIM23 pathway. Overexpression of Tim21, a regulatory subunit of the TIM23 complex, shifts the activity state of TIM23 from matrix translocation towards inner membrane insertion^31^,^32^, which we confirmed in *fmc1*Δ mitochondria (Fig. 3a,b; wild-type import results, confirmation of Tim21 overexpression and steady-state levels of other mitochondrial proteins in Supplementary Figs 4a–c and 6d,e). We found that Tim21 overexpression caused a substantial restoration of respiratory growth of the *fmc1*Δ mutant, while wild-type yeast was unaffected (Fig. 3c; Supplementary Fig. 4d). Likewise in human, overexpression of *TIMM21* led to a significant increase in the survival of *atp6-T8993G* NARP patient-derived cybrids (2.4-fold, *P*<0.05, Fig. 3d; Supplementary Data Set 1; confirmation of TIMM21 overexpression in Supplementary Fig. 1d), while wild-type cybrids were unaffected (Supplementary Fig. 1e). These observations support TIM23’s involvement in mutant rescue and attest to an evolutionarily conserved therapeutic effect of modulating mitochondrial protein sorting on ATP synthase dysfunction.

### Tim21 promotes OXPHOS biogenesis in a yeast disease model

**Figure 4:**
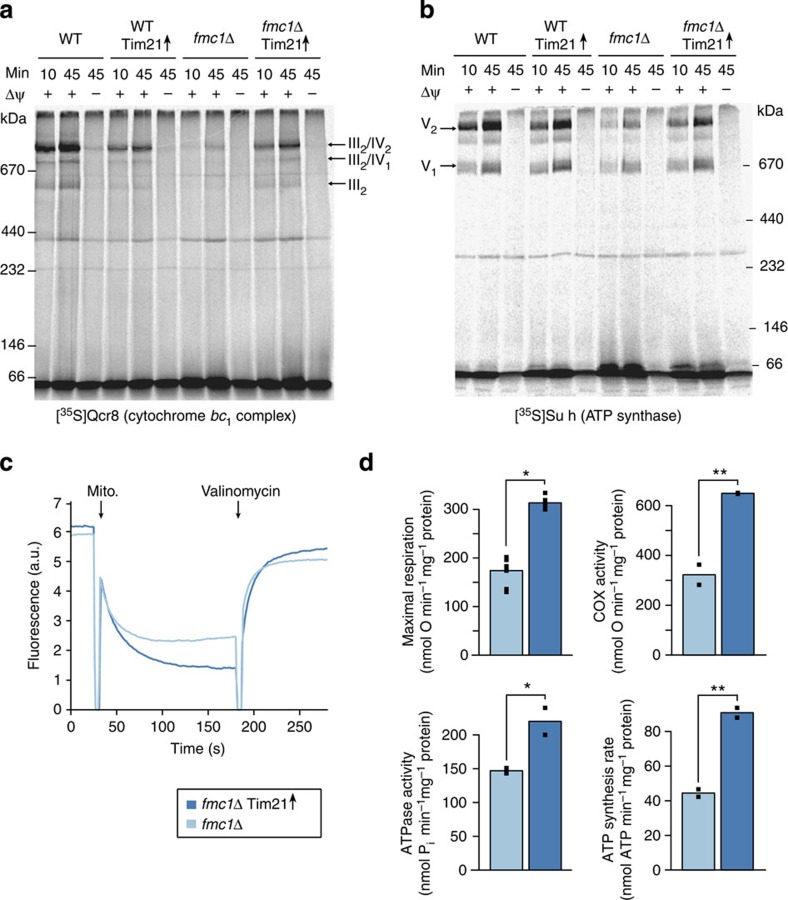
Modulation of mitochondrial protein sorting considerably restores the bioenergetic capacity of a yeast model of ATP synthase disorders. (**a**,**b**) Tim21 overexpression accelerates the incorporation of subunits Qcr8 and Su h into cytochrome *bc*_1_-containing respiratory chain supercomplexes and ATP synthase, respectively, in *fmc1*Δ mitochondria. Radiolabelled, *in vitro*-synthesized proteins were imported into isolated mitochondria and their assembly into their respective complexes (indicated with arrows) was followed using BN-PAGE and autoradiography. Δψ, mitochondrial inner membrane potential; III_2_/IV_2_, III_2_/IV_1_, III_2_: respiratory chain supercomplexes formed by complex III (cytochrome *bc*_1_) and IV (cytochrome *c* oxidase, COX); V_1_ and V_2_, monomeric and dimeric F_1_F_o_-ATP synthase (complex V), respectively. (**c**) Genetic modulation of mitochondrial protein sorting improves the mitochondrial inner membrane potential (Δψ) of *fmc1*Δ yeast. The Δψ of indicated mitochondria was monitored using a DiSC_3_(5) fluorescence quenching assay (see Methods). The difference in fluorescence before and after addition of valinomycin indicates the magnitude of Δψ of the analysed mitochondria. (**d**) Genetic modulation of mitochondrial protein sorting improves the bioenergetic capacity of *fmc1*Δ yeast. The following parameters were measured in isolated mitochondria: maximal/uncoupled respiratory rate (upper left), maximal/uncoupled cytochrome *c* oxidase (COX) activity with ascorbate/TMPD as a substrate (upper right), oligomycin-sensitive ATP hydrolysis using a colorimetric ATPase assay (lower left), and oligomycin-sensitive ATP synthesis using a luciferase assay (lower right). Bar height corresponds to median value of individual measurements displayed as points (both technical and biological replicates). Significance according to a *t*-test is represented as **P<0.01 or *P<0.05.

Another evolutionary parallel between yeast and human Tim21 is their interaction with OXPHOS complexes: the former physically couples respiratory chain supercomplexes to the TIM23 machinery^36^, and the latter facilitates the assembly of nuclear-encoded subunits into cytochrome *c* oxidase (complex IV)^30^. Notably, perturbations in complex IV biogenesis have been observed in ATP synthase disorders^10^,^12^. Moreover, we observed that Tim21 overexpression reduced the accumulation of matrix-localized aggregates composed of unassembled ATP synthase subunits in *fmc1*Δ mitochondria (Supplementary Fig. 4e). Altogether, these findings led us to investigate whether Tim21 could be facilitating the assembly of imported proteins into mature OXPHOS complexes in yeast. We indeed discovered pronounced improvements in OXPHOS complex assembly specific to our disease model (Fig. 4a,b; import controls in Supplementary Figs 5g and 8f,g). Our results demonstrate that Tim21 can promote the biogenesis of deficient OXPHOS complexes, and indicate that the connection between mitochondrial protein import and complex assembly could be an important target for alleviating mitochondrial dysfunction.

### Tim21 enhances ATP synthesis in disease model mitochondria

Finally, we investigated the impact of modulating TIM23-dependent mitochondrial protein sorting on the defective bioenergetic properties of *fmc1*Δ mitochondria that are also found in other mutants with deficient ATP synthase^8^,^9^,^10^,^11^,^12^. An improvement in respiratory chain activity in response to Tim21 overexpression was revealed by marked increases in both mitochondrial inner membrane potential (Δψ) and mitochondrial respiration (maximal uncoupled rates in whole cells and isolated mitochondria; Fig. 4c,d; Supplementary Fig. 5i; Supplementary Data Set 4). Consistent with the improvement in OXPHOS complex assembly, the specific activities of cytochrome *c* oxidase and ATP synthase were also significantly enhanced (Fig. 4d; latter as oligomycin-sensitive ATP hydrolysis, corroborated by in-gel ATPase activity in Supplementary Fig. 5f; Supplementary Data Set 4). These activity increases were reflected in the elevated steady-state levels of OXPHOS complexes (Supplementary Figs 5a–e and 8a–e). Most importantly, mitochondrial ATP synthesis was increased by greater than twofold (Fig. 4d). None of these bioenergetic properties were affected by Tim21 overexpression in wild-type yeast (Supplementary Fig. 5j; Supplementary Data Set 4). Taken together, our findings demonstrate that modulation of mitochondrial protein sorting via the TIM23 pathway alleviates not only a primary ATP synthase deficiency, but also an array of critical downstream phenotypes found in these disorders^10^,^12^,^15^,^16^.

### Conclusions and outlook

Our study reveals mitochondrial protein sorting as a promising new intervention point for the treatment of ATP synthase disorders. This constitutes the first demonstration that this fundamental process central to mitochondrial biogenesis and functionality can be fine-tuned via pharmacological or genetic intervention to suppress disease-associated phenotypes. While a more general inhibition of such a major pathway would likely be detrimental, the slight modulations applied here are beneficial in the context of mitochondrial dysfunction, just as in a recent study that improved survival of a mouse model of an ATP synthase disorder by inhibiting the mTOR signalling pathway^37^. That we were led to this precise, unexpected mode of action by a large-scale phenotypic screen and a genome-wide mutant profile attests to the power of system approaches for uncovering candidate intervention points. Our findings indicate that the therapeutic benefits of modulating protein sorting for ATP synthase deficiencies are conserved between yeast and humans. Other studies have indicated that moderately downregulating this pathway can help to maintain mitochondrial proteostasis under stressful conditions^33^,^34^,^38^, which along with future work may help to define how exactly the modulations we describe here exert their therapeutic effects. The numerous regulatory factors and mechanisms involved in mitochondrial protein sorting now present new avenues to explore therapeutic strategies for ATP synthase disorders. Due to the fundamental role of this pathway, the therapeutic potential of mitochondrial protein sorting could extend to a variety of diseases involving mitochondrial dysfunction.

## Methods

### Data analysis

All statistical analyses in this study were performed using R; the specific methods and packages are detailed in the appropriate sections.

### Chemical screening assay

This assay was performed as previously described^14^: 240 μl of exponentially growing *fmc1*Δ yeast, adjusted to an OD_600_ of 0.2, was spread homogeneously using sterile glass beads (~3 mm diameter) on a square Petri dish (12 × 12 cm) containing rich solid yeast-peptone-glycerol (YPG) medium (1% (w/v) BD Bacto yeast extract, 2% (w/v) BD Bacto peptone, 3% (v/v) glycerol). Sterile filters were placed on the agar surface, and 2.5 μl of each compound from the Prestwick chemical library dissolved in DMSO was applied to the filters, with DMSO separately applied as a negative control. The plates were incubated at 37 °C for 6 days and scanned using a Snap Scan1212 (Agfa).

### Yeast strain generation and *in vivo* assays

Growth assays with the yeast ATP synthase disorder model strain *fmc1*Δ (MC6) on rich glycerol media with NaPT (Fig. 1a) were carried out as previously described^14^. The strain background MC1 was used as wild type (*MATa ade2–1 his3–11,15 trp1–1 leu2–3,112 ura3–1* (*Δi E*^*R*^ *O*^*R*^); *fmc1*Δ/MC6 is the same genotype plus *Δfmc1::HIS3*)^8^. For Tim21 overexpression, we used the plasmid pYEP352-*TIM21*, a multicopy expression vector with an insert of the *TIM21* gene and its endogenous promoter and terminator regions^31^. Strains overexpressing Tim21 (and control strains carrying the empty vector pYEP352) were constructed by standard yeast plasmid transformation^39^, except that the heat shock step was omitted due to the thermosensitivity of the strains and instead, samples were left shaking gently (200 r.p.m.) overnight at room temperature (22 °C). Cells were plated on synthetic glucose media lacking uracil for selection using the *URA3* marker; the resulting transformants were then restreaked to obtain single colonies. For growth tests (Fig. 3c and Supplementary Fig. 4), strains were grown overnight at 30 °C in SD-Ura (1.7 g l^*−*1^ yeast nitrogen base without amino acids (BD Difco), 20 g l^*−*1^ glucose (Sigma), 1.92 g l^*−*1^ Yeast Synthetic Dropout Medium Supplement without Uracil (Sigma)). The next day, cells were either: (a) directly transferred to rich glycerol medium (YPG, 5 μl preculture in 100 μl medium) in a 96-well plate (NunclonΔ Surface) and grown at 36 °C in a TECAN GENios multiwell plate reader until saturation (48 h; liquid growth tests, Fig. 3c); or (b) diluted to OD_600_~0.5 in SD-Ura and grown for 5 hours to reach exponential phase (spot dilutions, Supplementary Fig. 4). The cultures were subsequently diluted to OD_600_=0.08 and then fivefold serially diluted in SGly-Ura (synthetic glycerol media lacking uracil as above, but with 3% glycerol (v/v) instead of glucose). Five microliters of each cell suspension were spotted on SGly-Ura agar plates that were then incubated for 6–7 days at 35 °C and scanned with a Canon CanoScan LiDE 600F. Growth rates (Fig. 3c) were estimated as the maximum slope of smooth fits of the raw data (local polynomial fit of order 2, bandwidth of 3 h) using the R/Bioconductor cellGrowth package.

### Chemical–genomic profiling

The homozygous and heterozygous yeast deletion pools were grown in YPD+25 mM HEPES (pH adjusted to 6.8) supplemented with 1% DMSO (compound vehicle, negative control) or 8.35 μM of NaPT for 5 and 20 generations, respectively, as previously described^21^. Genomic DNA extraction, PCR amplification of molecular tags and Genflex Tag16k array (Affymetrix) hybridization, washing and scanning were performed as previously described^21^.

### Analysis of chemical–genomic data

Each probe on the Genflex Tag16k array (Affymetrix), known as the Tag4 array, is represented by five replicate features^40^. Signal intensities were first log_2_-transformed. Next, each tag was summarized by the median intensity across all (mostly five) matching probes on the array. We then adjusted for overall signal intensity and PCR amplification of up and down tags as follows: For each pool, some tags typically do not yield a signal above background levels, either because the strain has vanished from the pool, it does not grow or it has acquired too many mutations and therefore does not hybridize to the array. Only tag intensities above 8 (a cutoff set from visual inspection of the intensity distributions as separating a low- from a high-intensity subpopulation) were considered above background. The intensity distributions of the up- and down-tag populations in each microarray were shifted by a separate constant so that all intensity distributions above background have equal medians. Differential strain enrichment between DMSO and NaPT treatment was determined using limma’s moderated t-test^41^ treating tag as an additive covariate to control for difference in hybridization efficiency between up and down tags. *P* values were corrected for multiple testing with the Benjamini–Hochberg method^42^, all using the R/Bioconductor package limma. The fold changes calculated can be found in Supplementary Data Set 2.

### Comparison to previously generated deletion sensitivity profiles

The *z*-score for every homozygous or heterozygous deletion strain was computed following the same procedure as in Hillenmeyer *et al*.^23^, that is, *z=(μ*_ctrl_*–μ*_exp_*)/σ*_ctrl_, where *μ*_ctrl_ and *μ*_exp_ are the means of the normalized log_2_ intensities in the DMSO controls and the NaPT (8.35 μM) experiments, respectively, and *σ*_ctrl_ is the sample standard deviation of log_2_ intensities in the controls. The *z*-scores for the data from this study can be found in Supplementary Data Set 2. These *z*-scores were then directly compared with those for 726 experiments comprising 332 compounds from profiles in ref. 23.

### Isolation of mitochondria from *Saccharomyces cerevisiae*

Wild-type cells (YPH499; *MATa ura3-52 lys2-801_amber ade2-101_ochre trp1-Δ63 his3-Δ200 leu2-Δ1* (ref. 43)) were grown in liquid rich glycerol medium (YPG) at 30 °C. *fmc1Δ* and corresponding wild-type cells were precultured at 28 °C in rich lactate medium (1% (w/v) BD Bacto yeast extract, 1% (w/v) BD Bacto peptone, 2% (w/v) Lactate, 1% (w/v) KH_2_PO_4_, 200 μM adenine, adjusted to pH 5.5 with KOH) and subsequently shifted to 37° for 20–24 h before harvesting. Cells transformed with plasmid pYEP352-*TIM21* or pYEP352 were precultured at 28 °C in liquid synthetic minimal medium (0.67% (w/v) yeast nitrogen base, 0.077% CSM amino acid mix minus uracil, 3% (v/v) glycerol, 0.2% (w/v) glucose) to ensure maintenance of the plasmids and then transferred to rich lactate medium at 37 °C for 20–24 h. *In vivo* NaPT treatment was performed using *fmc1*Δ and corresponding wild-type cells carrying the empty vector pYEP352. After a 24-h preculture in liquid synthetic minimal medium at 28 °C, 120 nM NaPT was added and the preculture was continued for another 24 h. Subsequently, cells were transferred to rich lactate medium containing 120 nM NaPT and grown at 37 °C for 20 h. Mitochondria were isolated by differential centrifugation^44^. For the bioenergetic measurements (respiration and ATP synthesis/hydrolysis assays) as well as for Supplementary Fig. 5d–f, a slightly different isolation protocol was used^45^.

### Import of precursor proteins into isolated mitochondria

For generation of radiolabelled Qcr8 and Su h, linear DNA templates with SP6 promoters were synthesized by PCR and transcribed *in vitro* using the mMESSAGE mMACHINE SP6 kit (Invitrogen, USA). mRNA was purified (MEGAclear kit, Invitrogen) and translated in reticulocyte lysate (Flexi Rabbit Reticulocyte Lysate System, Promega) in the presence of [^35^S]methionine. [^35^S]cytochrome *b*_2_-DHFR, [^35^S]cytochrome *b*_2_Δ-DHFR, [^35^S]Tom40 and [^35^S]AAC were synthesized by coupled *in vitro* transcription/translation using plasmid templates and the TNT SP6 Quick coupled kit (Promega) in the presence of [^35^S]methionine. [^35^S]cytochrome *b*_2_-DHFR is a fusion protein consisting of the N-terminal 220 amino acids of the *S. cerevisiae* cytochrome *b*_2_ precursor (including the mitochondrial import signals) fused to mouse dihydrofolate reductase^46^. On translocation via the TIM23 complex, the N-terminal presequence of this precursor is cleaved by the mitochondrial processing peptidase (MPP), resulting in an intermediate (i) form. Downstream of the N-terminal presequence, [^35^S]cytochrome *b*_2_-DHFR harbours a stop-transfer signal that prevents complete translocation of the protein into the matrix and promotes its integration into the inner mitochondrial membrane, followed by a second processing by the inner membrane protease and release of a soluble mature (m) form into the intermembrane space. [^35^S]cytochrome *b*_2_Δ-DHFR is similar to [^35^S]cytochrome *b*_2_-DHFR but lacks the stop-transfer signal (amino acids 47–65) and is therefore fully translocated across the inner membrane into the matrix and accumulates as the intermediate (i) processing form^47^. Recombinantly expressed cytochrome *b*_2_Δ-DHFR carries the same deletion of the stop-transfer signal as [^35^S]cytochrome *b*_2_Δ-DHFR but is based on a DHFR fusion of a shorter part of cytochrome *b*_2_ (amino acids 1–167). The resulting cytochrome *b*_2_Δ-DHFR fusion protein was expressed from the plasmid pUHE 73-1 in *Escherichia coli* strain BMH 71-18 and purified as previously described^48^. Protein import reactions typically contained 50 μg of isolated mitochondria (total protein amount) diluted in 100 μl import buffer (250 mM sucrose, 80 mM KCl, 5 mM MgCl_2_, 5 mM methionine, 10 mM KH_2_PO_4_, 10 mM MOPS–KOH, pH 7.2, 3% (w/v) bovine serum albumin, 2 mM NADH and 4 mM ATP). No bovine serum albumin was included in import reactions using recombinantly expressed *b*_2_Δ-DHFR. Reactions were supplemented with an ATP-regenerating system (20 mM creatine phosphate, 0.1 mg ml^*−*1^ creatine kinase) for the import and assembly of Qcr8, Su h, Tom40 and AAC. Samples were prewarmed to 25 °C, incubated with either CuCl_2_, NaPT or DMSO for 5–15 min where indicated, and import reactions were started by the addition of precursor proteins. Import of Tom40 was stopped by transferring the reactions to ice. Import reactions of all other precursors were terminated by the addition of AVO mix (8 μM antimycin A, 1 μM valinomycin, 20 μM oligomycin; final concentrations)^49^. Where indicated, non-imported precursors were removed by treatment with 50 μg ml^*−*1^ proteinase K (Roche). Mitochondria were washed with SEM buffer (250 mM sucrose, 10 mM MOPS, pH 7.2, 1 mM EDTA) and pelleted by centrifugation. Pellets were either solubilised in Laemmli buffer for analysis by SDS–PAGE or in digitonin buffer (20 mM Tris-HCl, pH 7.4, 0.1 mM EDTA, 10% (v/v) glycerol, 50 mM NaCl, 1% (w/v) digitonin, 1 mM PMSF (Roth)) for analysis by blue native electrophoresis (BN-PAGE). Radiolabelled proteins were detected by digital autoradiography and quantified using the ImageQuant(TM) software (GE Healthcare Life Sciences); quantifications for Fig. 2a,b can be found in Supplementary Data Set 3. Import of the recombinantly expressed *b*_2_Δ-DHFR (Fig. 2c) was assessed by western blotting using a polyclonal antibody directed against the DHFR moiety of the preprotein.

### Statistical analysis of import dynamics

For each substrate (Fig. 2a,b), three independent import time series were obtained (Supplementary Data Set 3). Within each series, the band intensities corresponding to the imported, protease-resistant proteins were normalized by dividing by the intensity for DMSO at the final time point. Next, a linear model with intercept 0 was fitted by least square regression in the presence or absence of NaPT, separately. The ratio of the fitted slopes represents how the rate of import was affected by the presence of NaPT. To assess statistical significance, the asymptotic likelihood ratio test was applied, comparing the goodness of fit of this model with two slopes against a null model assuming a common slope to both conditions (presence or absence of NaPT).

### BN-PAGE of isolated mitochondria

For BN-PAGE analysis^50^, mitochondrial extracts (starting from 40–100 μg mitochondrial proteins) solubilized with digitonin (digitonin to protein ratio of 10 g/g) were separated on NativePAGE 3–12% Bis-Tris gels (Invitrogen, USA) or on homemade blue native gradient gels (5–10%, 4–16.5% or 6–16.5% polyacrylamide). After electrophoresis, gels were either stained with Coomassie blue, incubated in a solution of 5 mM ATP, 5 mM MgCl_2_, 0.05% lead acetate, 50 mM glycine–NaOH, pH 8.4 to detect ATPase activity^51^, or transferred to nitrocellulose or PVDF membranes and analysed by western blotting^52^. Membranes were first incubated with peptide antibodies against Cox4, Atp4 or F_1_β, or polyclonal antibodies raised against cytochrome *b*. Subsequently, they were incubated with peroxidase-conjugated secondary antibodies at a 1:5,000 dilution (Promega or Sigma) and revealed using enhanced chemiluminescence.

### Membrane potential measurement

Mitochondria isolated from wild-type (YPH499) yeast or *fmc1*Δ and the corresponding wild type carrying Tim21 overexpression or empty plasmids were diluted in membrane potential assay buffer (0.6 M sorbitol, 0.1% (w/v) bovine serum albumin, 10 mM MgCl_2_, 0.5 mM EDTA, 20 mM KP_i_ pH 7.2; supplemented with 5 mM succinate and 5 mM malate for *fmc1Δ* and corresponding wild type) with or without 150 μM NaPT. The mitochondrial inner membrane potential was determined by measuring fluorescence quenching of the membrane potential-sensitive dye 3,3′-dipropylthiadicarbocyanine iodide [DiSC_3_(5)] using an Aminco-Bowman series 2 luminescence spectrometer (Thermo Spectronic) at room temperature with an excitation of 622 nm and an emission of 670 nm (ref. 53).

### Respiration rates

For whole-cell respiratory activity measurements, cells were resuspended in YP medium at 1 OD_600_ _nm_ ml^*−*1^ and oxygen consumption rates were measured with a Clark electrode. The reactions were started by adding 1% pure ethanol as the substrate. Uncoupled maximal respiratory rates were measured after adding 80 μM carbonyl cyanide 3-chlorophenylhydrazone (CCCP). For respiration measurements in isolated mitochondria, oxygen consumption rates were measured with a Clark electrode in the respiration buffer (0.65 M mannitol, 0.36 mM EGTA, 5 mM Tris-phosphate, 10 mM Tris-maleate, pH 6.8) as previously described^54^: mitochondria (0.15 mg ml^*−*1^) were placed in a 1.2-ml thermostatically controlled chamber at 28 °C in respiration buffer. Uncoupled maximal respiratory rates were measured in the presence of 4 mM NADH as substrate and 4 μM CCCP as uncoupler. The specific activity of cytochrome *c* oxidase was measured using a combination of 12.5 mM ascorbate and 1.4 mM *N,N,N',N*'-Tetramethyl-p-phenylenediamine dihydrochloride (TMPD) to reduce cytochrome *c* and in the presence of 4 μM CCCP as uncoupler. All of these measurements can be found in Supplementary Data Set 4.

### ATP synthesis and hydrolysis

For ATP synthesis rate measurements, mitochondria were incubated as for oxygen consumption experiments. The reaction was started by the addition of 4 mM NADH and 1 mM ADP and stopped by addition of 3.5% perchloric acid and 12.5 mM EDTA. The samples were then neutralized to pH 6.5 by the addition of KOH and 0.3 M MOPS. ATP was quantified in a luciferin/luciferase assay (Perkin Elmer) with an LKB bioluminometer. ATP hydrolysis rates were measured at pH 8.4 with a previously described procedure^55^. Oligomycin-sensitive ATP synthesis and hydrolysis rates were measured by adding oligomycin (20 μg mg^*−*1^ of protein) to estimate the relative amounts of fully assembled F_1_F_0_-ATP synthase. These measurements can be found in Supplementary Data Set 4.

### Statistical analysis of bioenergetics data

For measurements of respiration rates (whole-cell and isolated mitochondria), specific cytochrome *c* oxidase activity, and ATP synthesis and hydrolysis, statistical significance of the differences between strains carrying the Tim21 overexpression versus empty plasmid was assessed using a one-sided *t*-test assuming equal variance in both the groups. Two biological replicates were measured per strain. Technical replicates, which displayed lower variance than biological replicates, were summarized by their median value before testing. The individual measurements can be found in Supplementary Data Set 4.

### Human cell lines and culture conditions

The cybrid survival assays with NaPT treatment in Fig. 1b and Supplementary Fig. 1e were performed as previously described^14^: cell lines were first cultivated in high-glucose DMEM, after which the medium was removed and survival measurements were carried out in glucose-deprived DMEM supplemented with the indicated concentrations of NaPT, dihydrolipoic acid (DHLA;+control)^18^ or DMSO (− control); no antibiotics were used. Survival assays started with 3.5 × 10^4^ JCP239 eb13.13 (NARP *atp6-T8993G*) cells^18^ and 2 × 10^4^ JCP213 (control) cells being plated in 12-well plates containing DMEM with high glucose (4.5 g l^*−*1^) for 24 h. For each treatment condition, three wells were used. NaPT solutions in DMSO were diluted 1:1,000 in the medium. DHLA at 100 μM or the same volume of DMSO were used as positive and negative controls, respectively. After 6 days of incubation with the drugs, cell proliferation was estimated by cell counting using the Scepter (Millipore). Experiments were performed at least three times per condition and the values obtained can be found in Supplementary Data Set 1. Cell counts in each condition were expressed as a proportion of cell counts in DMSO control experiments (‘Percentage of live cells (relative to untreated)’). For statistical analysis, an analysis of variance model was fitted with intercept at 100% growth, and significance of the effect for each concentration was assessed by the *t*-test using the R package lmtest. *TIMM21* overexpression experiments were performed using JCP239 eb13.13 and JCP213 cybrid cell lines transduced with lentiviral constructs overexpressing *TIMM21* (pLOC-Tim21 OHS5897-101185497) or RFP as control (pLOC-RFP-control OHS5832), both provided by Open Biosystems. HIV1-based lentiviruses were produced in HEK293FT cells according to ref. 56. Both cell lines were transduced with a multiplicity of infection of 1. At 4 days post transduction, cells carrying the different constructs were seeded separately in six-well plates (10^5^ cells per well) containing DMEM with glucose. After 24 h of growth, three wells for each line were used to estimate the total number of cells per well using a C6 flow cytometer (Accuri). The medium was removed from remaining wells and cells were further incubated with DMEM without glucose (as described above). After 6 days of incubation, cell proliferation/survival was estimated by cell counting using a C6 flow cytometer (Accuri). Experiments were performed at least three times per condition and three different wells were counted for each line in each experiment; the values obtained can be found in Supplementary Data Set 1. Cell counts in each condition were expressed as a proportion of the cell counts following the first 24 h of growth in Fig. 3d. To confirm TIMM21 overexpression, 10 μg protein of cell lysates from JCP239 cells transduced with overexpression constructs were separated on SDS–PAGE and subjected to western blot analysis using anti-TIMM21 (anti-C18orf55) affinity-purified rabbit polyclonal antibodies (Proteintech Europe) and revealed by enhanced chemiluminescence.

## Author contributions

R.S.A., M.v.d.L., M.Bo., J.P.d.R., M.Bl., C.V., S.D.-C., C.F., C.A. and L.M.S. designed the research; S.S. and R.P.S. generated the chemical–genomic profiles, which were analysed by J.G., R.S.A. and R.P.S.; R.S.A., M.Bo. and K.v.d.M. performed the import, assembly and biochemical assays; R.S.A., E.C., C.V., E.S.F., F.S., J.E., M.Bo. and K.v.d.M. performed the *in vivo* yeast work; R.S.A. and R.K. generated yeast strains; C.F. performed the electron microscopy with the help of R.S.A. and both analysed it with the help of J.G.; C.V. and F.S. assayed cybrid survival in the presence of NaPT; S.D.-C. and F.C. performed the cybrid transduction and survival assays; J.G., S.D.-C., and C.V. analysed all the cybrid data; S.D.-C., F.C. and M.Bo. performed the assays of mitochondrial bioenergetics; R.S.A., M.v.d.L., J.P.d.R., M.Bl., C.A. and L.M.S. supervised the research; and R.S.A., M.v.d.L., J.P.d.R. and L.M.S. wrote the manuscript.

## Additional information

**How to cite this article:** Aiyar, R. S. *et al*. Mitochondrial protein sorting as a therapeutic target for ATP synthase disorders. *Nat. Commun.* 5:5585 doi: 10.1038/ncomms6585 (2014).

## Supplementary Material

Supplementary Information Supplementary Figures 1-8, Supplementary Methods and Supplementary References

Supplementary Data 1 Raw counts of surviving atp6-T8993G and control cybrids in response to NaPT treatment or TIMM21 overexpression (Figs. 1b, 3d).

Supplementary Data 2 Systematic chemical-genomic profiles derived from the yeast homozygous and heterozygous deletion collection treated with NaPT (Fig. 1). The z-scores as computed in Hillenmeyer *et al*.3, fold changes as described in Methods, and gene annotation according to the Saccharomyces Genome Database are provided, in tab-delimited format.

Supplementary Data 3 Intensities of bands detected by digital autoradiography from SDSPAGE gels corresponding to radiolabeled mitochondrial proteins imported via the TIM23 complex (Fig. 2a-b), in tab-delimited format.

Supplementary Data 4 Measurements of mitochondrial respiration (whole-cell and isolated mitochondria), cytochrome c oxidase-specific activity, ATP synthesis, and ATP hydrolysis in fmc1Δ yeast overexpressing Tim21, in tab-delimited format.
