# Supplementary material for: Mitochondrial protein sorting as a therapeutic target for ATP synthase disorders
Source: Nat Commun. 2014 Dec 18;5:5585. doi: 10.1038/ncomms6585 (PMC4284804; doi:10.1038/ncomms6585)
Supplement: Supplementary Information — Supplementary Figures 1-8, Supplementary Methods and Supplementary References [file ncomms6585-s1.pdf]

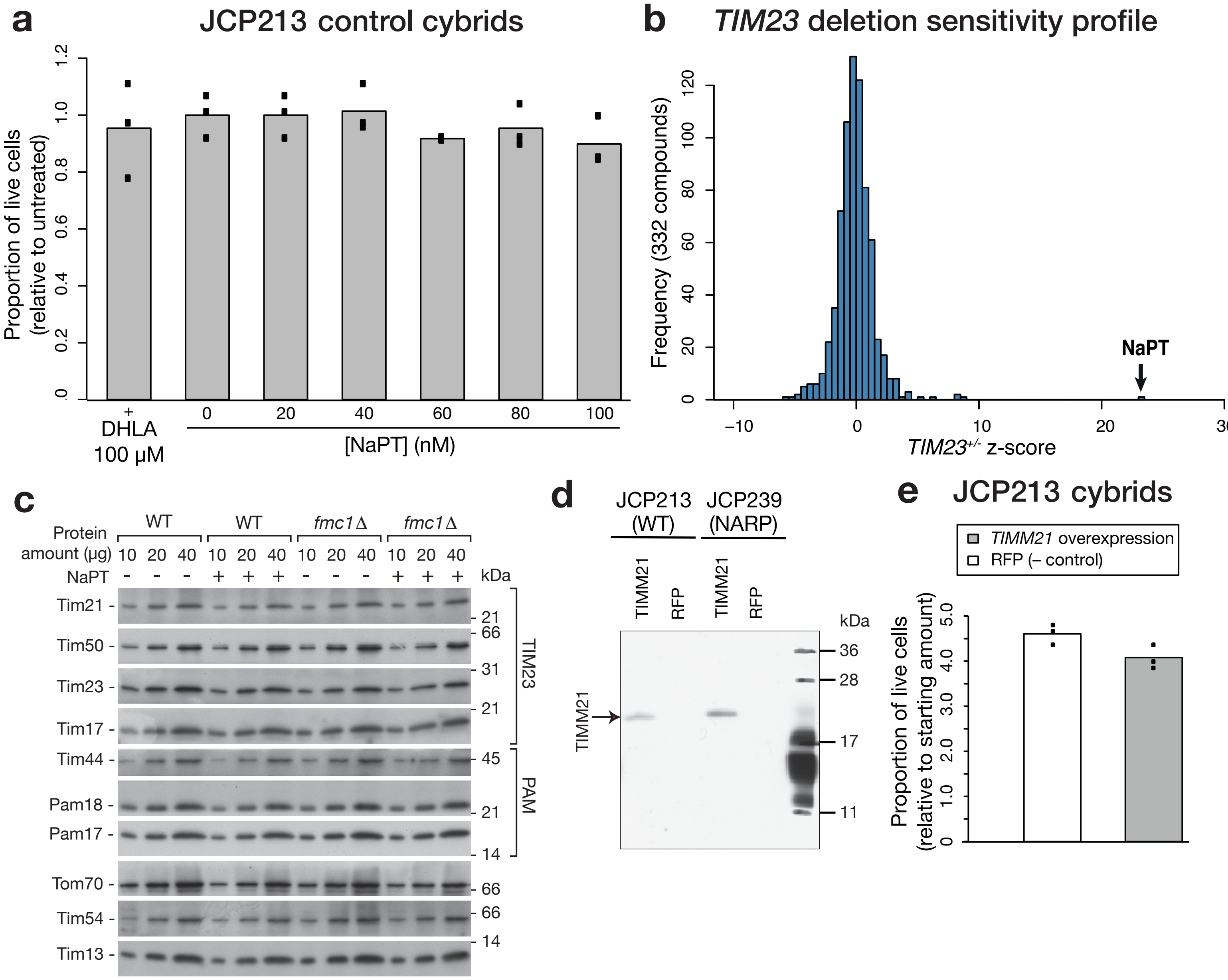

Supplementary Figure 1. Control experiments on patient-derived cybrids and *in vivo* effects of NaPT in yeast.

(**a**) NaPT treatment does not affect control cybrids with wild-type mtDNA. Cybrids (JCP213; ref. 1; see Methods) were treated with the indicated concentrations of NaPT for 6 days in glucose-deprived medium, and the number of live cells was measured using a Scepter, revealing no statistically significant changes in cell numbers. Data shown is from three replicates per condition, indicated by points; height of the bar represents the mean. DHLA, + control currently in clinical trials for mitochondrial encephalopathies (ref. 2).

(**b**) Frequency distribution of the *TIM23*$^{+/-}$ mutant z-scores (see Methods) from 726 published chemical genomic profiles (ref. 3) across 332 compounds compared to the score obtained for NaPT.

(**c**) Confirmation of *TIMM21* overexpression in TIMM21 transfected control (WT) and NARP (*atp6-T8993G*) patient-derived cybrids versus RFP negative control transfected versions of these cybrids via western blot.

(**d**) Overexpression of *TIMM21*, the human ortholog of yeast *TIM21*, does not affect survival of control cybrids in glucose-deprived medium. JCP213 wild-type cybrids were transduced with lentiviral particles carrying *TIMM21* and GFP, or RFP alone as a negative control. After 6 days, surviving cells were counted using flow cytometry and the survival rates were computed relative to the starting amount following glucose removal. Individual replicates are displayed as points; height of the bar represents the mean. No significant difference in survival rates was detected using the Wilcoxon test.

(**e**) NaPT treatment does not alter the levels of TIM23 and import motor subunits. Steady-state protein levels of subunits of the TIM23 machinery (Tim21, Tim50, Tim23, Tim17), the TIM23-associated import motor PAM (Tim44, Pam18, Pam17), as well as control proteins (Tom70, subunit of the general preprotein translocase of the outer membrane [TOM complex]; Tim54, subunit of the carrier translocase of the inner membrane [TIM22 complex]; Tim13, intermembrane space chaperone) of mitochondria isolated from wild-type (WT) or *fmc1Δ* cells grown in the presence or absence of 120 nM NaPT revealed by SDS-PAGE and western blotting.

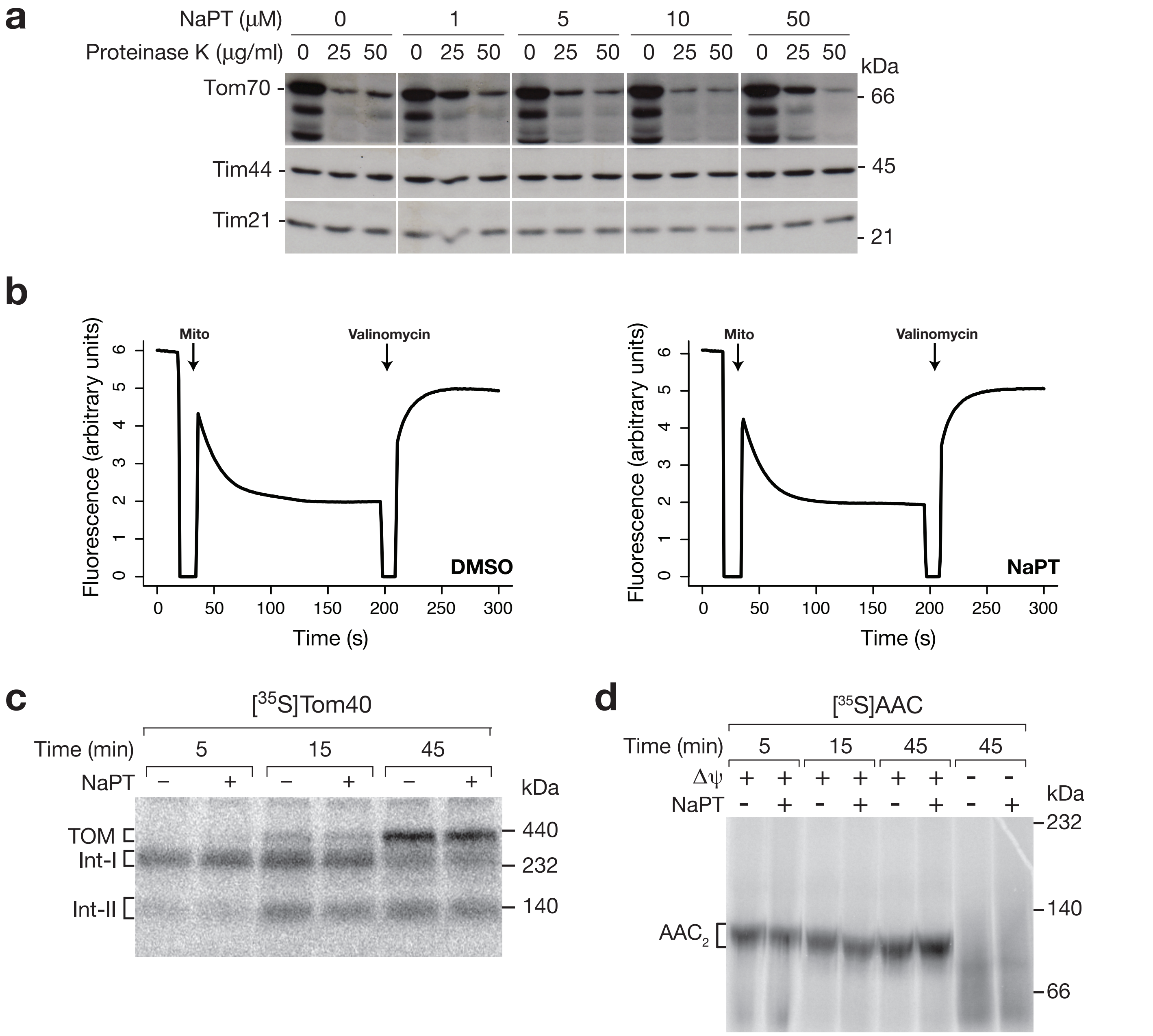

Supplementary Figure 2. Controls verifying that the effects of NaPT on mitochondrial protein import are specific to TIM23.

(**a**) NaPT does not disrupt mitochondrial membrane integrity. Mitochondrial protein import rates can be adversely affected by perturbing membrane integrity. To determine whether NaPT affects membrane integrity, wild-type mitochondria were incubated with NaPT at the indicated concentrations, followed by proteinase K treatment and western blotting for proteins in different mitochondrial compartments (Tom70 - outer membrane; Tim21 - inner membrane, exposed to intermembrane space; Tim44 - matrix). When mitochondrial membranes are intact, Tom70 is the only protein sensitive to proteinase K treatment, protease sensitivity of Tim21 would show a rupture of the outer membrane, whereas degradation of Tim44 by the protease would indicate that the inner membrane is also damaged.

(**b**) NaPT does not disrupt the generation of a membrane potential across the inner mitochondrial membrane. Since preprotein import via TIM23 is a membrane potential ($\Delta\psi$)-dependent process, we measured $\Delta\psi$ using a fluorescent dye (DiSC3(5)) that is taken up by mitochondria in a $\Delta\psi$-dependent manner in the presence of DMSO (left) or 150 μM NaPT dissolved in DMSO (right). The difference in fluorescence between the stabilisation post-addition of mitochondria (Mito) and the stabilisation post-addition of valinomycin, a potassium ionophore that uncouples $\Delta\psi$, indicates the magnitude of the membrane potential. This value is not affected by NaPT treatment. Data are representative of 5 independent experiments.

(**c, d**) NaPT does not affect import and assembly of outer membrane β-barrel proteins via the TOM and SAM complexes or of inner membrane metabolite carrier proteins via the TOM and TIM22 complexes. (**c**) Import and assembly of radiolabelled Tom40 precursor in the presence or absence of 150 μM NaPT analyzed by blue native electrophoresis. Int-I = Intermediate I (Tom40 precursor bound to SAM complex); Int-II = Intermediate II (Tom40-Tom5); TOM = mature TOM complex of 400 kDa. (**d**) Import and assembly of radiolabelled ADP/ATP carrier (AAC) in the presence or absence of 150 μM NaPT analyzed as in (c). AAC_2, dimeric, mature AAC; $\Delta\psi$, membrane potential across the inner mitochondrial membrane. Precursors of outer membrane β-barrel proteins, inner membrane metabolite carrier proteins with internal targeting signals and matrix- or inner membrane-targeted, presequence-carrying proteins all initially pass the outer membrane via the TOM complex and are then handed over to distinct downstream import pathways. Thus, the observed selective inhibition of the import of presequence-carrying preproteins, but not other classes of precursor proteins cannot be explained by a general impairment of import, *e.g.*, at the level of the TOM complex.

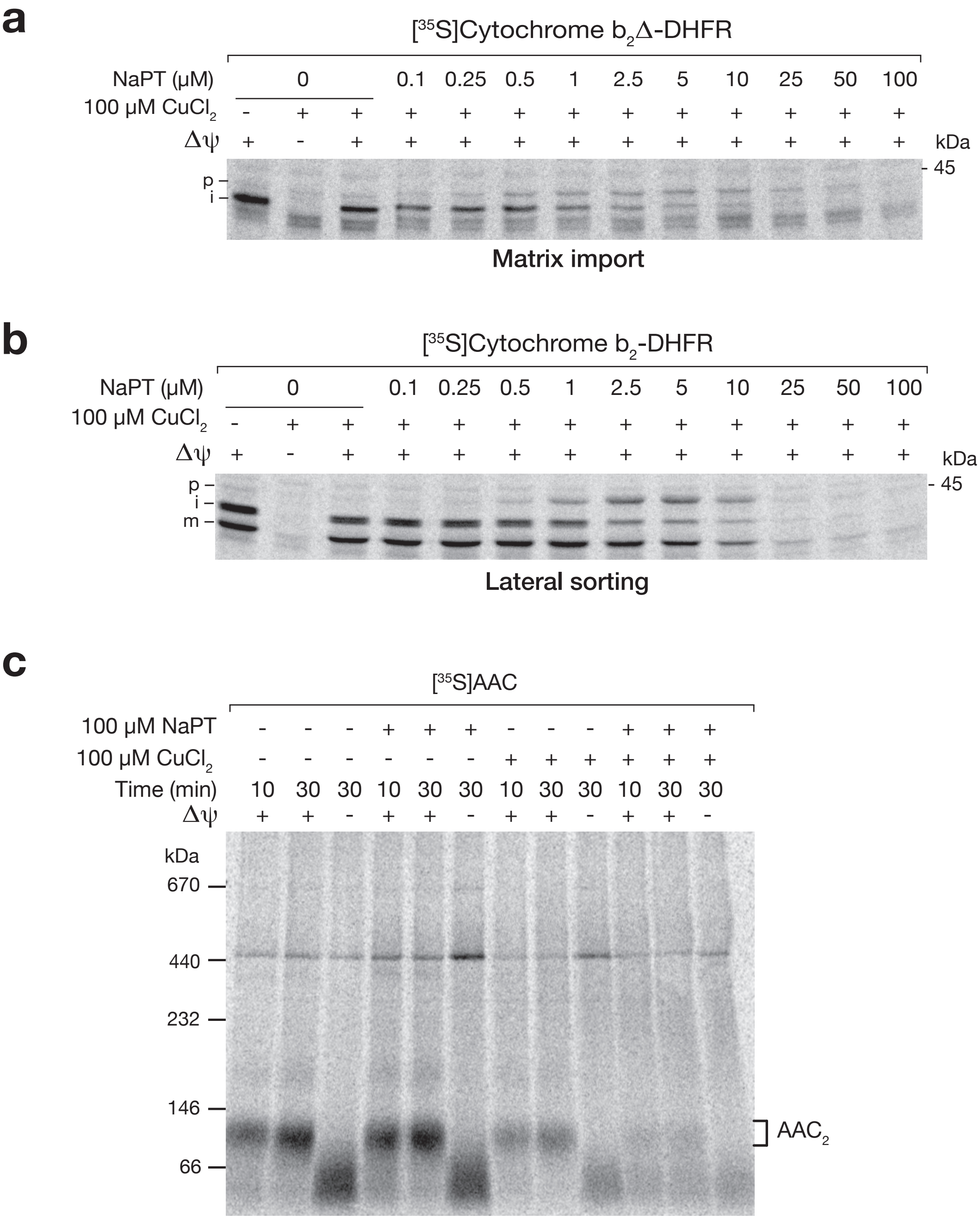

Supplementary Figure 3. Overlapping effects of NaPT and Cu^{2+} on mitochondrial protein sorting.

(a, b) Copper chloride alone inhibits both matrix translocation and lateral sorting via TIM23. The former effect is heightened in combination with NaPT, while in the latter case, although NaPT alone enhances lateral sorting (Fig. 2b), its combination with copper inhibits it. Mitochondria were pre-treated with indicated amounts of NaPT and $CuCl_2$ for 10 min followed by addition of indicated radiolabelled precursors and further incubation for 15 min. Non-imported precursors were digested with proteinase K and samples were analyzed by SDS-PAGE and digital autoradiography. $\Delta\psi$, mitochondrial membrane potential; p, precursor; i, intermediate processing form; m, mature form.

(c) Copper chloride alone inhibits import of metabolite carriers via the TIM22 pathway, and this effect is exacerbated in combination with NaPT. Radiolabeled ADP/ATP carrier (AAC) was incubated with isolated mitochondria pretreated with or without NaPT and/or $CuCl_2$. Mitochondria were solubilized with digitonin and analyzed by BN-PAGE and digital autoradiography.

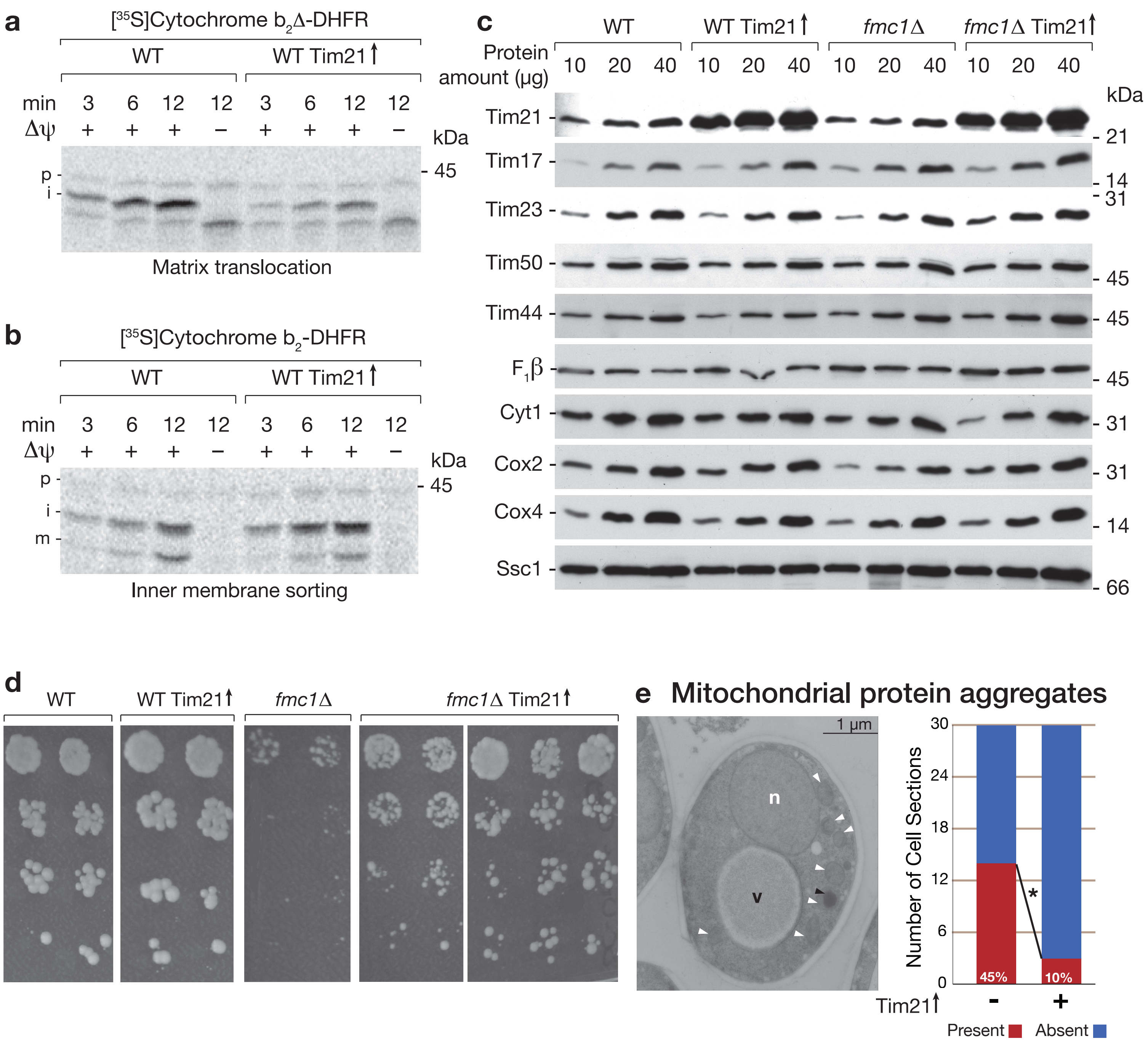

Supplementary Figure 4. Properties of *fmc1*Δ and wild-type yeast overexpressing Tim21.

(**a,b**) Tim21 overexpression induces NaPT-like modulation of TIM23-mediated mitochondrial protein sorting. Confirmation of differential modulation of TIM23-mediated import in the wild-type corresponding to *fmc1*Δ yeast, in accordance with a published study (ref. 4; assays as in Figs. 2 and 3, panels a,b).

(**c**) Steady-state levels of mitochondrial proteins revealed by western blots using the indicated antibodies on isolated mitochondria subjected to SDS-PAGE, including a verification of increased levels of the Tim21 protein upon overexpression.

(**d**) Tim21 overexpression improves growth of *fmc1*Δ yeast on solid synthetic glycerol media. Exponentially growing strains from Fig. 3c were diluted to OD_{600}=0.08, followed by serial fivefold dilutions and spotting onto synthetic glycerol media lacking uracil. Repeats correspond to biological replicates from the transformation reaction. Scans were made following 6 days of incubation at 35°C. WT, isogenic wild-type strain for *fmc1*Δ mutant; Tim21↑ , carrying *TIM21* overexpression plasmid; other strains carry corresponding empty plasmid.

(**e**) Tim21 overexpression reduces inclusion body formation in *fmc1Δ* mitochondria. In the absence of the assembly factor Fmc1, the α and β subunits of F_1-ATP synthase cannot be assembled and thus form large mitochondrial aggregates, visible as electron-dense material in the image (indicated with the black arrowhead). n, nucleus; v, vacuole; white arrowheads indicate mitochondria. Electron micrographs were scored for the presence of inclusion bodies (IBs) in cell sections, revealing a reduction by Tim21 overexpression. * indicates significant difference (*P*=0.03, Fisher's exact test for count data).

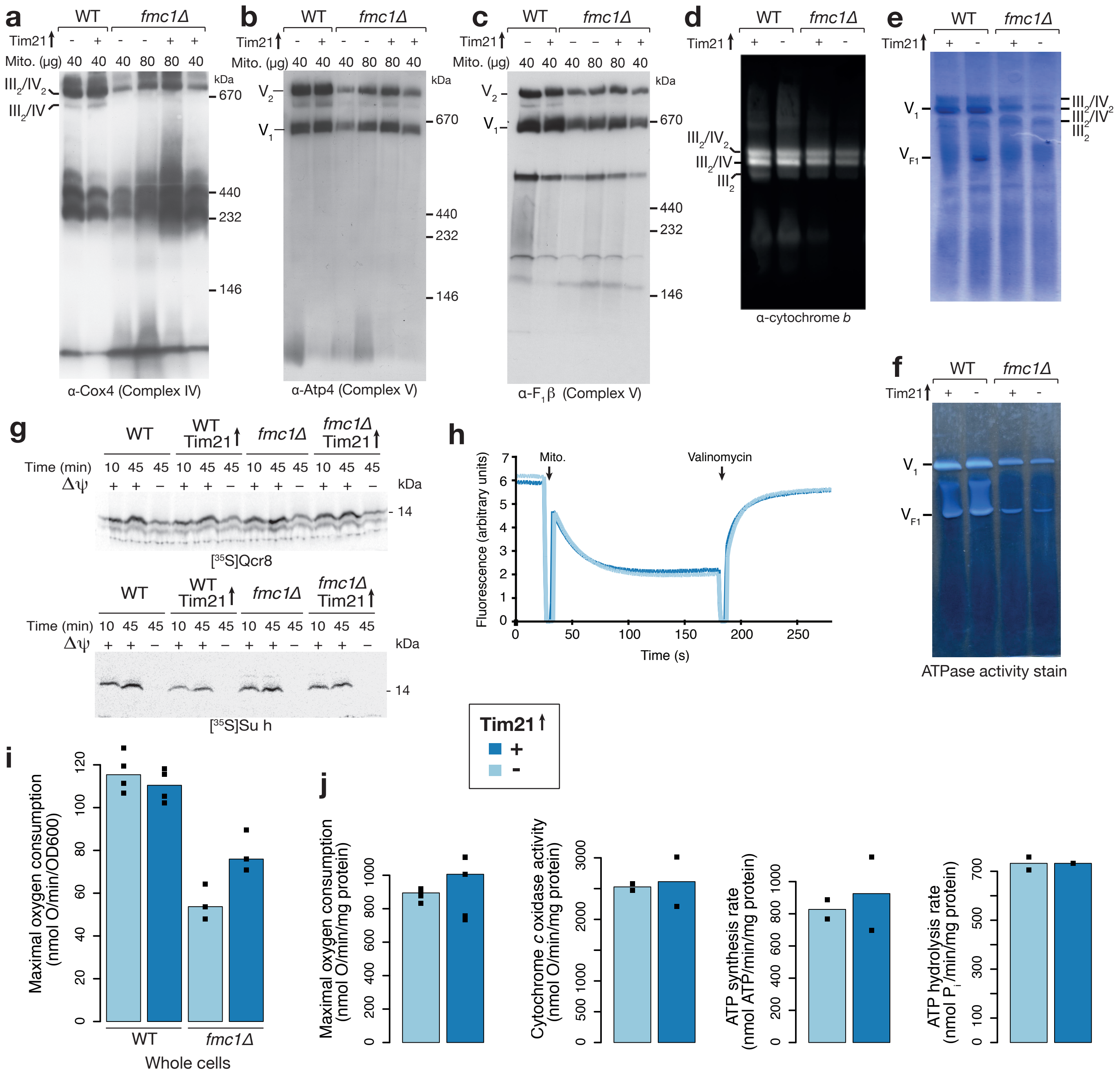

Supplementary Figure 5. Tim21 overexpression increases abundance of deficient OXPHOS complexes and mitochondrial respiration in *fmc1Δ* yeast, but does not affect bioenergetic properties of wild-type mitochondria.

In panels **a-f**, isolated mitochondria from the strains indicated were solubilized with digitonin and subjected to blue native gel electrophoresis (BN-PAGE, see Methods). OXPHOS complexes are indicated as follows: III_2/IV_2, III_2/IV_1, III_2: respiratory chain supercomplexes formed by complex III (cytochrome bc_1) and IV (cytochrome c oxidase, COX); V_1 and V_2, monomeric and dimeric F_1F_o-ATP synthase (complex V), respectively; V_{F1}, F_1 sector of ATP synthase.

(**a-d**) Steady-state levels of OXPHOS complexes III (cytochrome *b*), IV (Cox4), and V (Atp4, $F_1\beta$) as detected by western blots using the indicated antibodies.

(**e**) BN-PAGE Coommassie stain showing OXPHOS complexes.

(**f**) ATPase activity was assayed in-gel by incubating the native gel in an ATP buffer; inorganic phosphate released during ATP hydrolysis forms a white precipitate in the presence of lead.

(**g**) Import controls for OXPHOS assembly assays using SDS-PAGE analysis of the samples used for BN-PAGE in Fig. 4a,b.

(**h**) Mitochondrial membrane potential in wild-type yeast overexpressing Tim21. The Δψ of the indicated mitochondria was monitored using a $DiSC_3(5)$ fluorescence quenching assay.

(**i**) Whole-cell maximal (*i.e.*, uncoupled) respiration rates using ethanol as a substrate in *fmc1Δ* and its corresponding wild-type measured at the time of mitochondrial isolation for measurements in panel (j) and Fig. 4d.

(**j**) Wild-type yeast with the same genetic background as *fmc1Δ* carrying TIM21 overexpression or empty plasmids were assayed for their maximal/uncoupled respiratory capacity using isolated mitochondria (first panel), maximal/uncoupled cytochrome c oxidase activity with ascorbate/TMPD as a substrate (second), oligomycin-sensitive ATP synthesis using a luciferase assay (third), and oligomycin-sensitive ATP hydrolysis using a colorimetric ATPase assay (last) (corresponds to data shown for *fmc1Δ* yeast in Fig. 4d). Bar heights correspond to median values of individual measurements displayed as points (both technical and biological replicates). No significant differences ($P<0.05$) were detected using a t-test for these assays in response to Tim21 overexpression.

a Figure 2a

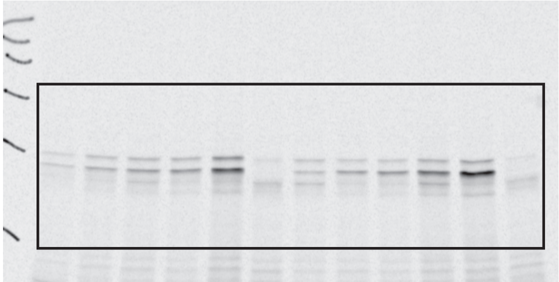

Autoradiography

b Figure 2b

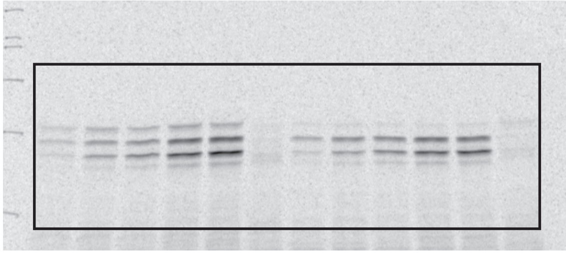

Autoradiography

c Figure 2c

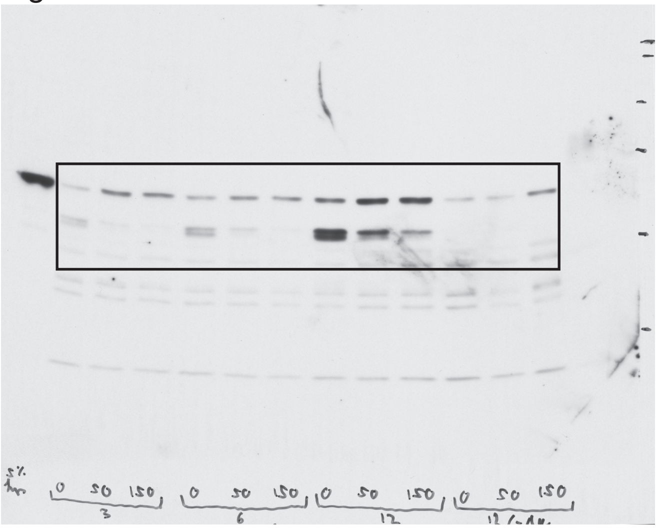

IB: DHFR

d left: Supplementary Figure 4a; right: Figure 3a

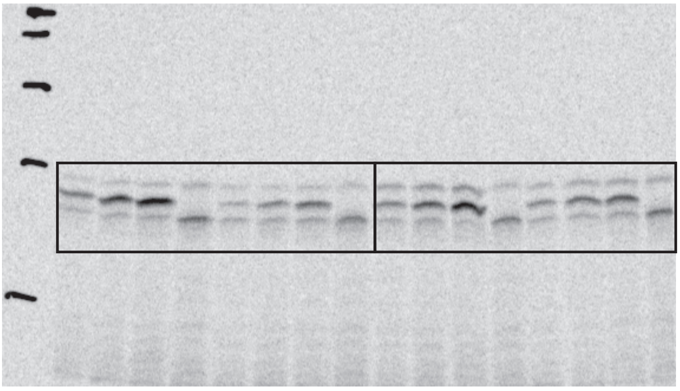

Autoradiography

e left: Supplementary Figure 4b; right: Figure 3b

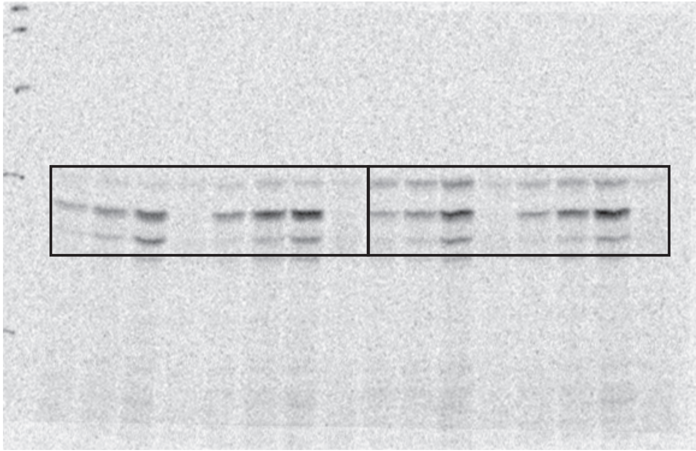

Autoradiography

Supplementary Figure 6. Original scans of western blots and gels presented in Figs. 2 and 3 and Supplementary Figure 4.

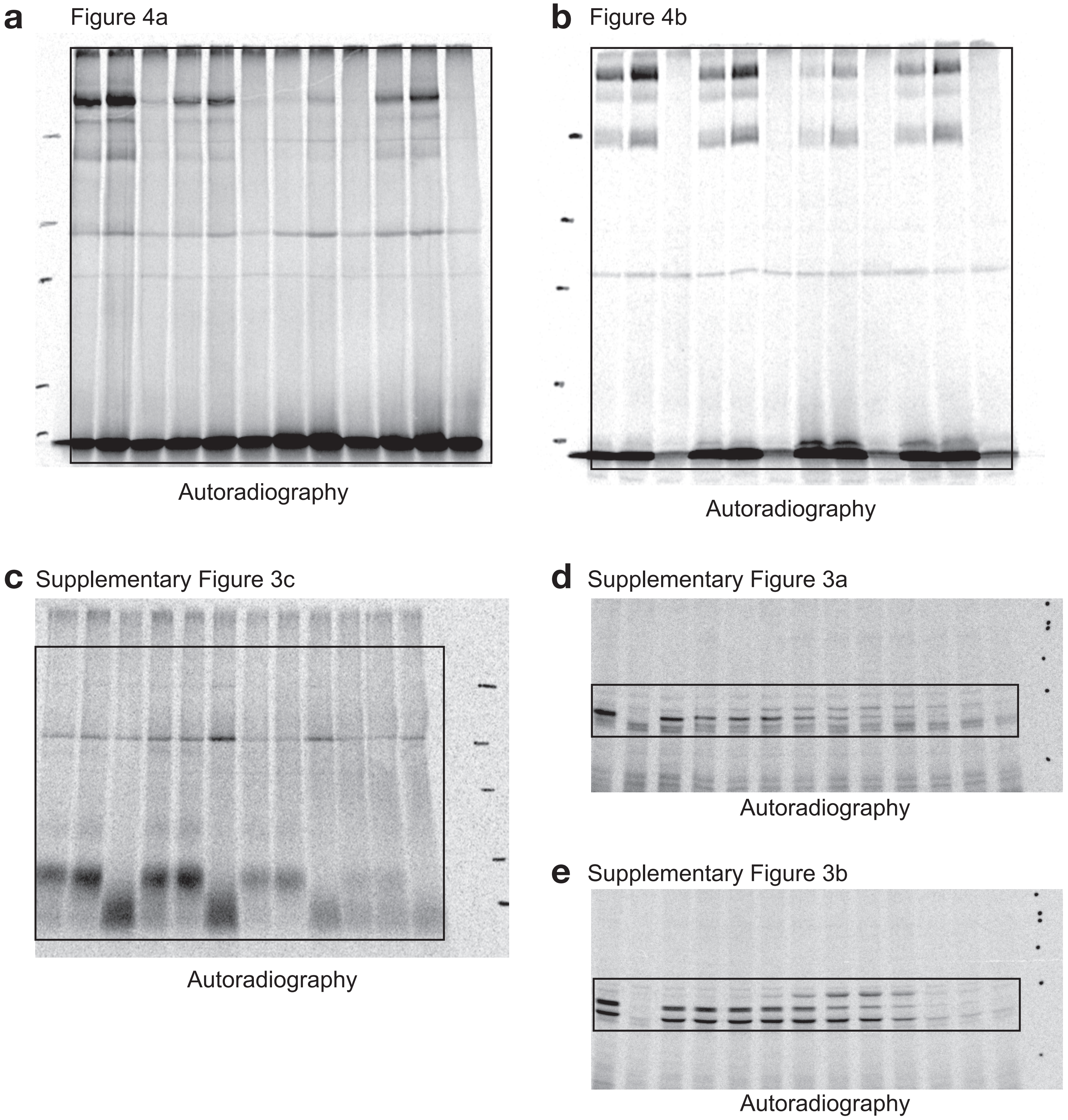

Supplementary Figure 7. Original scans of western blots and gels presented in Fig. 4 and Supplementary Fig. 3.

a Supplementary Figure 5a

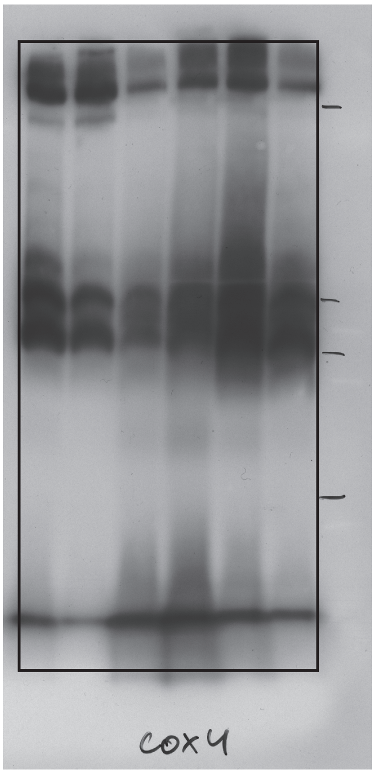

IB: Cox4

b Supplementary Figure 5b

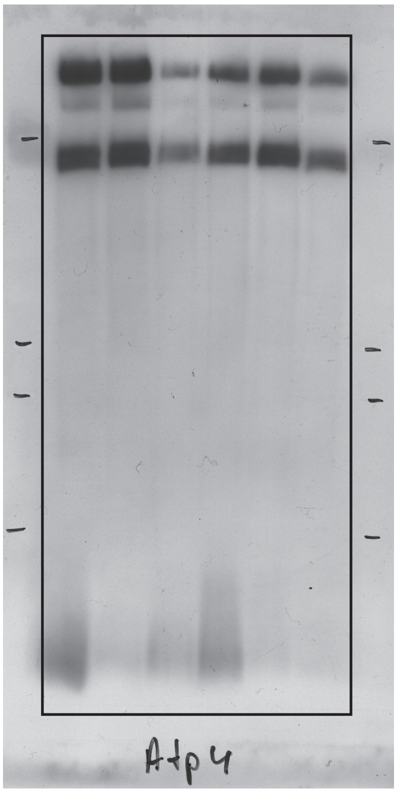

IB: Atp4

c Supplementary Figure 5c

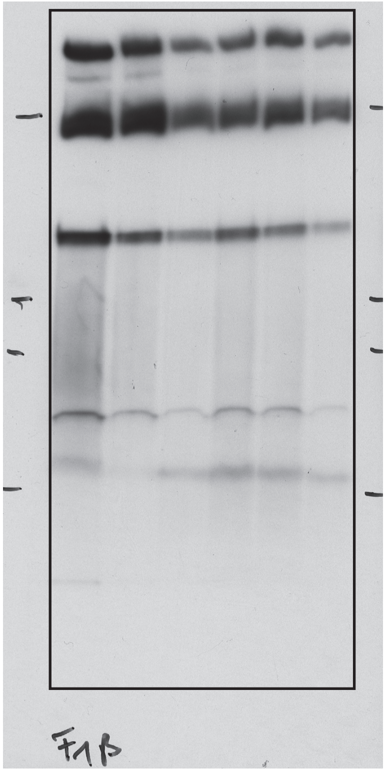

IB: $F_1\beta$

d Supplementary Figure 5e

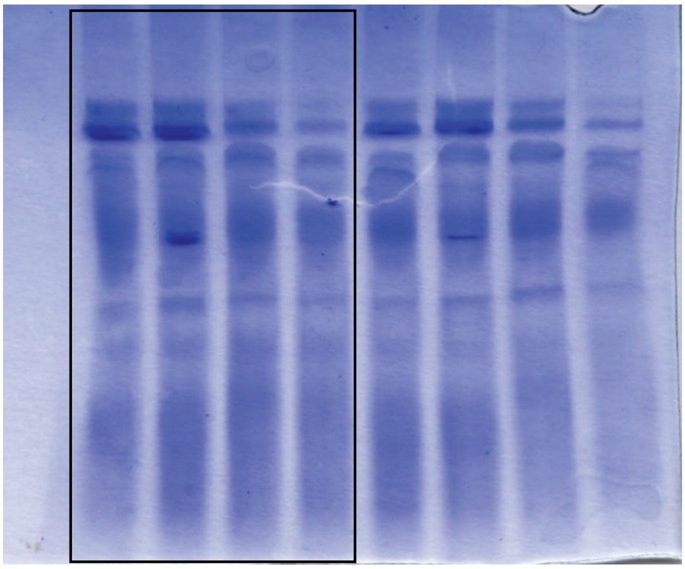

Coommassie stain

e Supplementary Figure 5f

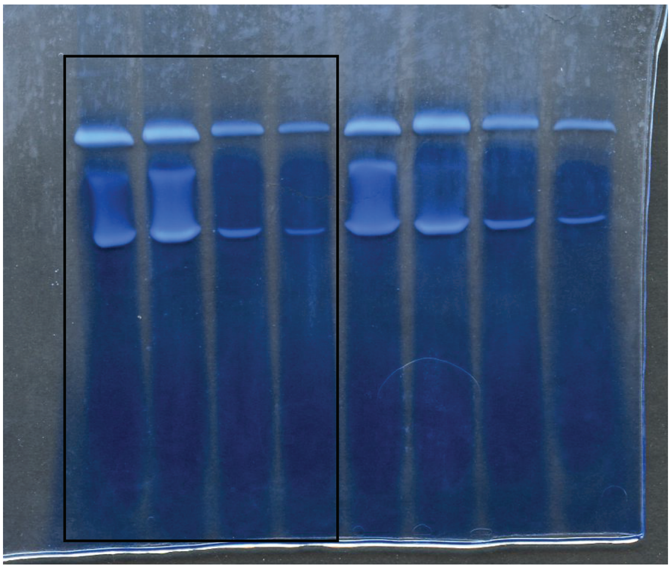

ATPase activity stain

f Supplementary Figure 5g upper panel

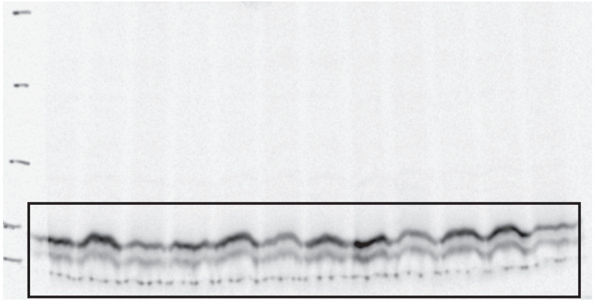

Autoradiography

g Supplementary Figure 5g lower panel

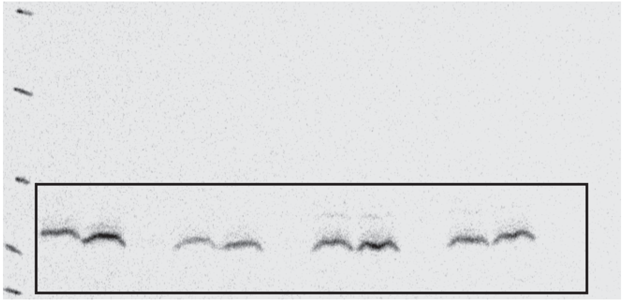

Autoradiography

Supplementary Figure 8. Original scans of western blots and gels presented in Supplementary Fig. 5.

Supplementary Methods

Membrane integrity assessment

Membrane integrity (Supplementary Fig. 2a) was assessed by incubating isolated wild-type mitochondria in SEM buffer supplemented with 0.5% DMSO or varying concentrations of NaPT (1-50 µM) dissolved in DMSO for 15 min at 25°C while shaking at 600 rpm. Samples were then subdivided into 50 µl aliquots and treated with varying concentrations (25 and 50 µg/ml) of proteinase K for 15 min on ice. Protease digestion was stopped by adding 1 µl of 0.2 M PMSF and incubating for 10 min on ice. Mitochondria were washed with SEM buffer and pelleted by centrifugation. Pellets were solubilized in Laemmli buffer and analysed by SDS-PAGE and western blotting using a peptide antibody against Tom70 (outer membrane) and holo antibodies against Tim21 (inner membrane, exposed to the intermembrane space), and Tim44 (matrix).

Electron microscopy

For electron microscopy analysis (Supplementary Fig. 4e), strains MC1 and MC6 (*fmc1Δ* and corresponding wild-type) carrying either pYEP352-*TIM21* or the empty vector pYEP352 were precultured overnight in SD-Ura at 30°C, followed by a second preculture in SGly-Ura to preserve the plasmids and a main culture in YPG (the latter 2 for 20 hours at 35°C).

Yeast cells were cryoimmobilised by high-pressure freezing with Leica EMPACT-2 (Leica Microsystems, Vienna, Austria). Freeze substitution of the cells was done using a freeze substitution device EM-AFS2 (Leica Microsystems, Vienna, Austria). The freeze substitution solution used contained 0.2% uranyl acetate, 0.1% glutaraldehyde and 1% water dissolved in anhydrous acetone; samples were substituted at -90°C for 50 hours. The temperature was then increased at a rate of 5°C per hour up to -45°C, followed by 5 hours of incubation at -45°C. The samples were rinsed with acetone three times for 10 min each, followed by lowicryl HM20 (Polysciences, Warrington, PA, USA) infiltration at 45°C with 25% lowicryl in acetone for 2 hours, 50% lowicryl for 2 hours and 75% lowicryl for 2 hours. The samples were then left in 100% lowicryl for 12 hours and in fresh 100% lowicryl for 2 hours before onset of polymerization. UV polymerization was applied for 48 hours at -45°C and then the temperature was increased to 20°C at a rate of 5°C per hour and the samples were left exposed to UV at room temperature for 48 hours. Thin sections (70 nm) were cut with a Leica Ultracut UCT microtome (Leica Microsystems, Vienna, Austria), and collected on Formvar-coated, palladium-copper slot grids. The sections were post-stained using uranyl acetate and lead citrate, and viewed using a CM120 BioTWIN electron microscope (FEI, Eindhoven, Netherlands) operating at 100 kV. Digital acquisitions were made with a KeenView CCD camera (Olympus Soft Imaging Solutions, Münster, Germany).

Supplementary References

1. Mattiazzi, M. *et al.* The mtDNA T8993G (NARP) mutation results in an impairment of oxidative phosphorylation that can be improved by antioxidants. *Hum. Mol. Genet.* **13,** 869–879 (2004).
2. Couplan, E. *et al.* A yeast-based assay identifies drugs active against human mitochondrial disorders. *Proc. Natl. Acad. Sci.* **108,** 11989–11994 (2011).
3. Hillenmeyer, M. E. *et al.* The Chemical Genomic Portrait of Yeast: Uncovering a Phenotype for All Genes. *Science* **320,** 362–365 (2008).
4. Chacinska, A. *et al.* Mitochondrial Presequence Translocase: Switching between TOM Tethering and Motor Recruitment Involves Tim21 and Tim17. *Cell* **120,** 817–829 (2005).
